# Substantial capacitance found in the roots of 2 contrasting conifer species

**DOI:** 10.1093/plphys/kiaf116

**Published:** 2025-05-07

**Authors:** Christopher McCarthy, Ibrahim Bourbia, Timothy Brodribb

**Affiliations:** School of Natural Sciences, University of Tasmania, Private Bag 55, Tas, Hobart 7001 Tasmania, Australia; School of Natural Sciences, University of Tasmania, Private Bag 55, Tas, Hobart 7001 Tasmania, Australia; School of Natural Sciences, University of Tasmania, Private Bag 55, Tas, Hobart 7001 Tasmania, Australia

## Abstract

High rates of photosynthesis require abundant water delivered to the canopy to replace water lost to transpiration. In addition to water drawn immediately from the soil, stem capacitance has been identified as an additional water source, particularly during transient transpiration states. However, little information is available about the potential of roots to contribute to plant capacitance because methodological constraints have made it challenging to quantify root capacitance. In this study, we present a method to measure the water storage capacity of the root system and assess its contribution to daytime transpiration. We used an optical dendrometer to obtain in situ measurements of water potential and transpiration in 2 contrasting conifer species, Oyster Bay pine (*Callitris rhomboidea*) and Monterey pine (*Pinus radiata)*, allowing us to quantify diurnal changes in plant water deficit. We employed a modified flow meter to gauge the rehydration kinetics of the below-ground and above-ground systems separately. We observed that root capacitance is a major supplier to the water demands during transient changes in transpiration for both species. Notably, the total below-ground capacitance exceeded the above-ground capacitance in *C. rhomboidea*, while the 2 capacitances were similar in *P. radiata*. Our findings highlight the importance of measuring and including below-ground capacitance in hydraulic models to accurately predict diurnal plant water status and stomatal behavior.

## Teaser

Above- and below-ground water storage measurements in 2 contrasting conifer species indicate root capacitance contributes to the water demands during transient changes in transpiration.

## Introduction

The enduring success of plants in dry, terrestrial environments hinges on their adaptive strategies to mine, store, and transport water. The diverse array of water management strategies exhibited by land plants is highlighted by the structural innovations that optimize water delivery to photosynthetic tissues in leaves, while being constrained by a myriad of environmental pressures. One generality that emerges from variation in water transport characteristics among species is that greater efficiency of the hydraulic pathway has been associated with higher rates of photosynthesis (Hubbard et al. 2001; Brodribb and Feild 2010). This association exists because a steady water supply is needed to balance the water loss from transpiration through the stomata during assimilation (Wong et al. 1979).

The flux of water flowing to the leaves depends on the water potential (Ψ) gradient from a source, usually the soil, to the leaf (Tyree 1988). However, changes in the physiology of the plant which increase water delivery are associated with costs such as building more xylem or increasing the Ψ gradient, which can lead to issues like water transport failure due to xylem cavitation and embolism (Tyree and Sperry 1989).

One strategy for improving water availability to leaves while reducing costs in water transport is for plants to store water. Water stored inside the plant can be released during episodes of increased evaporative demand thereby buffering the leaf against rapid changes in Ψ (Meinzer 2003; Meinzer et al. 2008; Scholz et al. 2011; Hao et al. 2013). Hydraulic capacitance, typically defined as the amount of water that can be extracted from storage per change in water potential (Zimmermann and Steudle 1979; Edwards and Jarvis 1982; Scholz et al. 2011), provides a transient water supply that is not subject to the substantial hydraulic resistance incurred at the soil-to-root barrier, by drawing from internal water storage (Goldstein et al. 1998). This not only decreases the net resistance to water supplied to the canopy but also fortifies the hydraulic system against potentially damaging conditions by moderating abrupt xylem pressure drops that can lead to cavitation (Brodribb et al. 2021), particularly during rapid transpiration increases due to environmental changes (Hao et al. 2013; Grossiord et al. 2020). In addition to reducing the risk of cavitation, drawing water from internal storage rather than from the soil provides a more direct and lower resistance water source; this dampens the water potential drop caused by sudden increases in evaporative demand giving stomata additional time to adjust transpiration to restore plant water potential homeostasis (Mcculloh et al. 2014)

In species known for large water storage ability, capacitance has been observed to provide a substantial proportion of water for daily transpiration needs under both well hydrated and dry conditions. Certain cacti and succulent species exemplify the use of capacitance in this way, and only continue to thrive in dry environments by relying exclusively on stored water and stringent stomatal control (Nobel and Goldstein 1992; Nobel et al. 1992). Interestingly, even in less hostile environments, many temperate and Mediterranean-adapted gymnosperms have also been shown to rely on stored water to supplement their transpiration demands throughout the day (Loustau et al. 1996; Maherali and DeLucia 2001; Phillips et al. 2003). The estimated contribution of stored water to the total water used varies widely among plants, from close to zero some herbaceous crops (Bourbia and Brodribb 2023) to 60% in the subtropical palm tree *Sabal palmetto* (Holbrook and Sinclair 1992). The wide variability of estimates of the contribution of stored water to daily transpiration highlights the variability between species, but an additional source of variation may be the diversity of methods employed to measure capacitance.

The most common technique for estimating capacitance contribution involves measuring the water release properties from a sample of sapwood (usually bored from the stem), against the corresponding change in water potential (Holbrook, 1995). By knowing the capacitance of a sapwood sample, it is possible to scale up to the whole tree to estimate how much water is released from storage during periods of high transpiration (Phillips et al. 2003). Other approaches use sap flow measurements from the crown and base of a tree to detect the lag in flow between water leaving the stem and water entering the stem from the soil, and this lag is assumed to be due to stem capacitance (Goldstein et al. 1998; Meinzer et al. 2003, 2004; Scholz et al. 2008).

The overwhelming majority of research on the influence and functionality of capacitance in trees has been centered on the stem and leaves, with minimal consideration given to the potential for substantial water storage in the root system. However, there are several reasons to suggest that roots could provide a substantial capacitance function. Firstly, root tissue typically accounts for a substantial portion of a plant's total biomass across various forest types (Bolte et al. 2004; Huang et al. 2021). This is especially true during a plant's sapling stage (Cao and Ohkubo 1998). Secondly, roots typically contain collapsible structures such as the cortex, which could release large quantities of water when the plant's water potential gradient decreases (Cuneo et al. 2021). Indeed, several studies have observed substantial shrinkage of roots during periods of declining water potential (Huck et al. 1970; Cole and Alston 1974; Faiz and Weatherley 1982). These studies collectively underscore the important role that root systems might play in water storage and management within plants, underscoring that roots are an essential component of plant water relations that warrants further investigation.

Here, we hypothesize that root capacitance in tree species could provide as much or more water for transpiration than the canopy due to the large amount of collapsible biomass underground. In this paper, we use a combination of approaches to measure root capacitance in 2 conifer species, Oyster Bay pine (*C. rhomboidea)* and Monterey pine (*P. radiata).* We choose these 2 species as conifers have been reported to exhibit high capacitance in their stems (Waring and Running 1978; Čermák et al. 2007; Mcculloh et al. 2014). Additionally, these species represent contrasting ends of the water management spectrum; *C. rhomboidea* has dense wood and tough xylem while *P. radiata* has lower wood density and more cavitation-vulnerable xylem (Jansen et al. 2012; Larter et al. 2017; Johnson and Brodribb 2023). Using a rehydration method, which enables us to characterize the individual contributions of canopy and root capacitance, we quantify the magnitude of root capacitance and its contribution to daytime transpiration in these 2 species.

## Results

### Characterizing typical diurnal water relations

Diurnal patterns of plant water potential and transpiration suggested substantial daily charging and discharging of capacitance was occurring.

**Figure 1.**
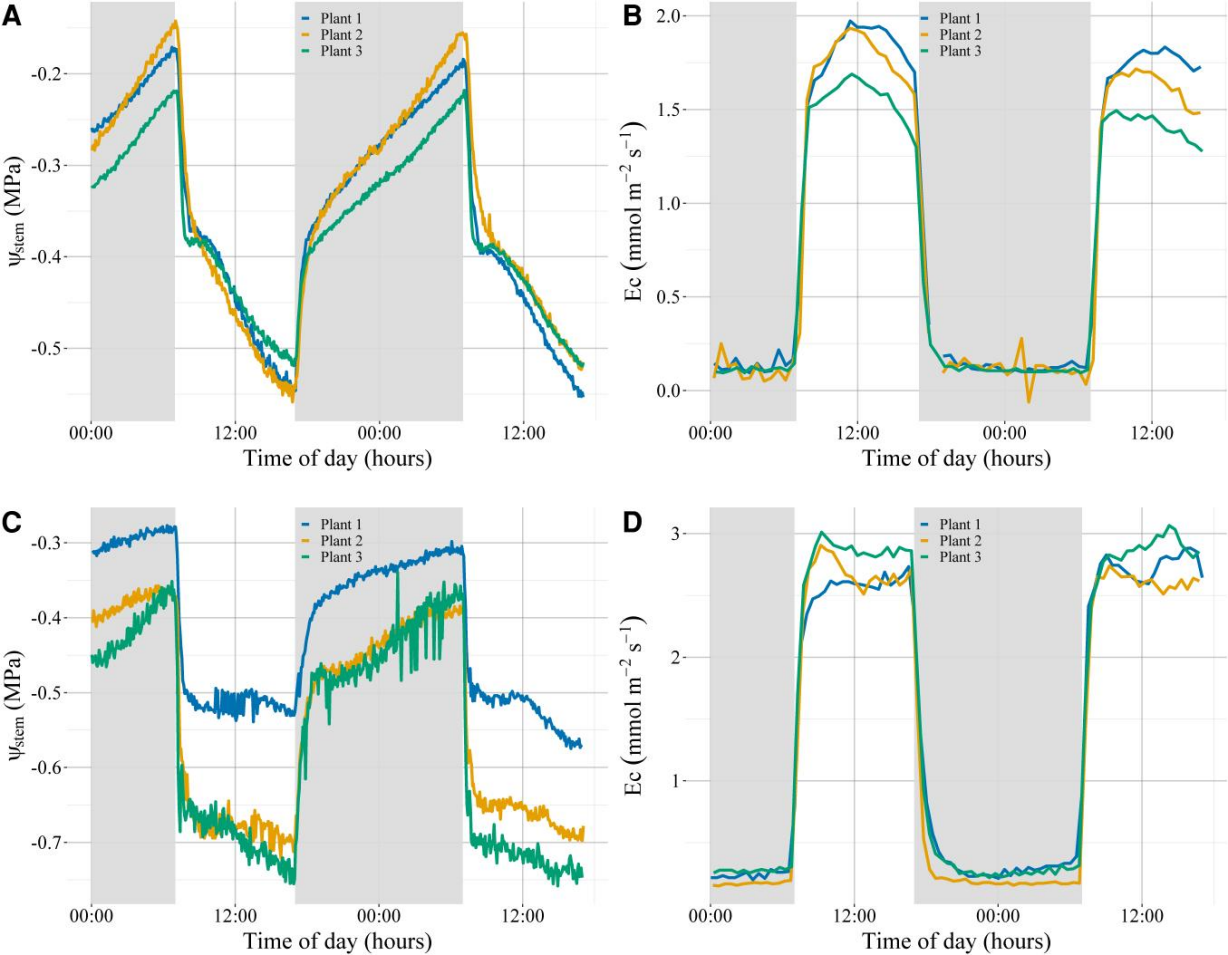
Diurnal pattern of Ψ_Stem_ and transpiration in *C. rhomboidea* and *P. radiata* over 2 well-watered consecutive days. Continuous Ψ_Stem_ measurements for *C. rhomboidea*  **(A)** and *P. radiata*  **(C)** were taken every 250 s using an optical dendrometer (*n* = 3 for both species). Diurnal patterns of *E*_c_ taken in parallel with Ψ_Stem_ data on the same *C. rhomboidea*  **(B)** and *P. radiata*  **(D)**. Dark gray sections indicate the night-time period.

**Figure 2.**
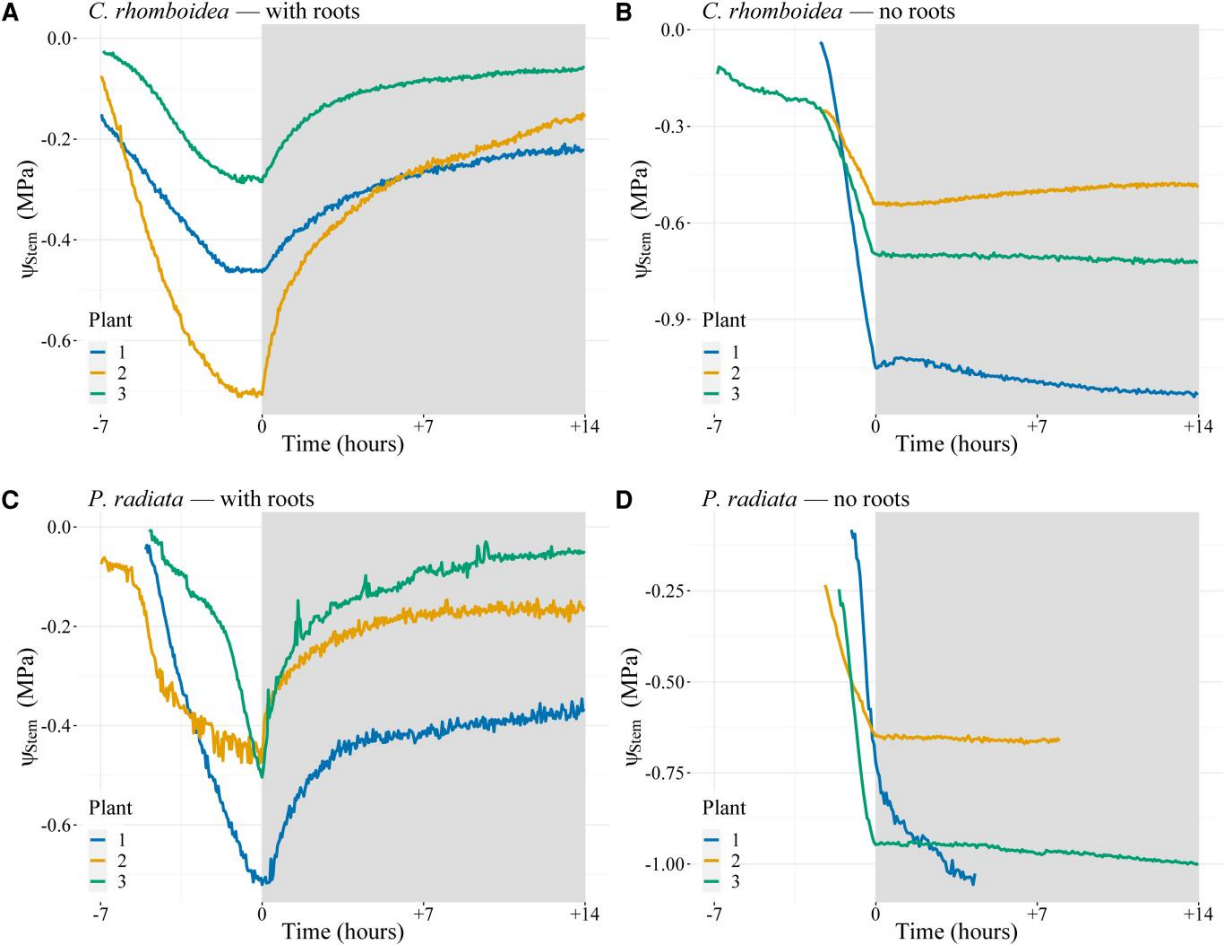
Stem water potential (ψₛₜₑₘ) rehydration response over time with and without roots in *C. rhomboidea* and *P. radiata.* Ψ_Stem_ of bare-rooted plants of *C. rhomboidea*  **(A)** and *P. radiata*  **(C)** during a day night cycle. White shading represents daytime conditions while gray shading represents the period plants (*n* = 3 for each treatment) were covered to prevent transpiration. Removal of roots in *C. rhomboidea*  **(B)** and *P. radiata*  **(D)** prevented any recovery of Ψ_Stem_ during the nontranspiring night period. Time 0 represents when the plant material was covered to prevent transpiration.

For both species, once the lights were turned on at 07:00 h, Ψ_Stem_ rapidly declined as *E*_c_ sharply increased. *E*_c_ for *C. rhomboidea* continued to rise until it peaked at midday, after which it slowly declined until the lights were turned off at 17:00. After the initial sharp decline, Ψ_Stem_ in *C. rhomboidea* briefly plateaued for 30 min before continuing to substantially decline throughout the day, despite previous work showing root conductance as well as root to mesophyll hydraulic conductance remains constant through the day (Bourbia et al. 2022; Bourbia and Brodribb 2024). *E*_c_ for *P. radiata* appeared to plateau after the sharp increase immediately following the lights being turned on and remained relatively constant until the lights were turned off at 17:00 (Figs. 1 and 2).

For both species, once the lights turned off at 17:00h, *E*_c_ rapidly declined to near zero after 1 h, yet the recovery of Ψ_Stem_ followed a much slower trajectory. Rehydration of the branchlets/needles followed 2 phases: a more rapid initial recovery in the first hour, followed by a more gradual increase that continued throughout the night (Fig. 1). In both species, the slow phase of Ψ_Stem_ recovery suggested a substantial proportion of water may have been drawn from capacitance during the day, which was replenished slowly overnight. The fact that rehydration continued throughout the 15 h night period despite a stable *E*_c_ close to zero suggests a substantial water flow into and out of capacitance during the diurnal cycle.

### Ψ_stem_ recovery with and without a root system

We tested whether the source of water leading to gradual nocturnal Ψ_Stem_ relaxation was uniquely from the soil, or partially due to water redistribution within the plant, by monitoring Ψ_Stem_ while subjecting plants to a day night cycle with and without soil. In soil free plants Ψ_Stem_ dropped rapidly during daytime transpiration but was able to largely recover when transpiration stopped in both species, with a mean recovery of 79% ± 8% toward initial Ψ_Stem_ in *C. rhomboid*ea and 74% ± 20% in *P. radiata* (Fig. 2). When the same experiment was conducted on the above-ground portion of the plants with roots completely excised, Ψ_Stem_ declined during the day but was not able to recover after transpiration declined in either species. This suggested that a substantial proportion of nocturnal rehydration of Ψ_Stem_ in these cases was coming from a local supply from water storage in the roots.

### Above- and below-ground capacitance

**Figure 3.**
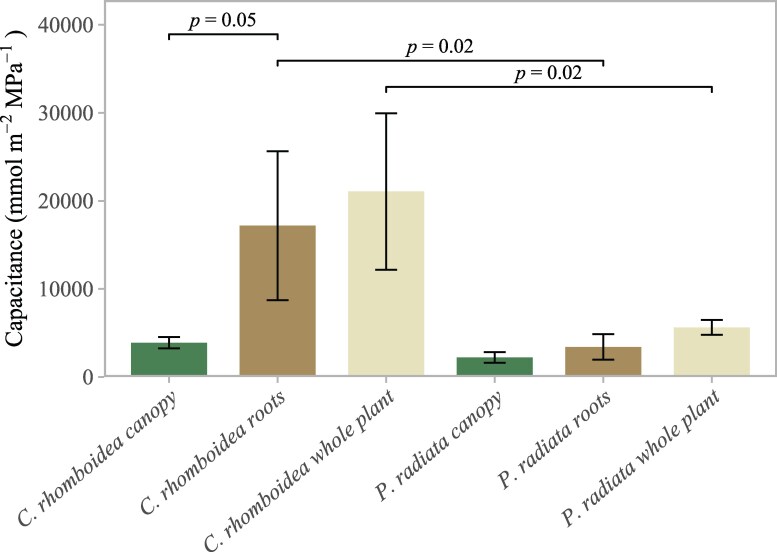
Root, canopy, and whole-plant capacitance in *C. rhomboidea* and *P. radiata*. Mean (±SD) absolute values of capacitance for *C. rhomboidea* and *P. radiata* (*n* = 3 for both species) separated by canopy (green) and roots (brown) and the whole plant (cream). Differences in roots and whole-plant contributions between *C. rhomboidea* and *P. radiata* were significant (Student's *t*-test, *P* < 0.05 for both comparisons). Difference in roots and canopy contributions for *C. rhomboidea* were not significant (Student's *t*-test, *P* > 0.05).

Based on rehydration data from excised whole root systems of 3 individuals, the mean capacitance of roots of *C. rhomboidea* was 17.2 ± 8.5 mol m^−2^ MPa^−1^. This was greater than the mean value for the above-ground tissue in *C. rhomboidea* of 3.8 ± 0.6 mol m^−2^ MPa^−1^ (Fig. 3).

*P. radiata* plants showed a similar trend in terms of higher capacitance in roots 3.4 ± 1.5 mol m^−2^ MPa^−1^ compared with above-ground tissue 2.2 ± 0.6 mol m^−2^ MPa^−1^, but both were substantially lower than the very high mean capacitance evident in *C. rhomboidea* roots.

### Capacitance as a source of water for rapid changes in transpiration

**Figure 4.**
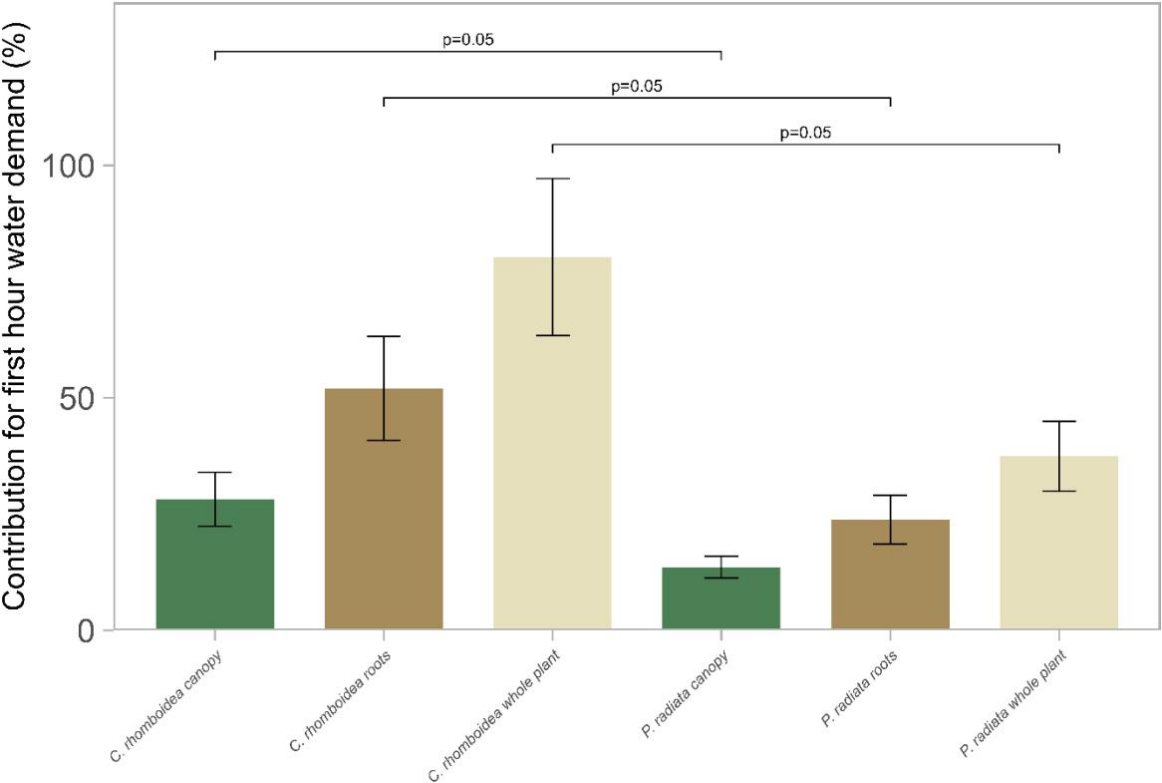
Contribution of root, canopy, and whole-plant water storage to first hour water demand in *C. rhomboidea* and *P. radiata*. Percentage (±SE) of total transpired water sourced from internal storage in the canopy (green), root (brown), and the combination of both (cream) for *C. rhomboidea* and *P. radiata* during the first hour of transpiration (07:00–08:00 h). Values represent means calculated from *E*_c_ and C measurements (*n* = 3 for both species). Differences in canopy, root, and whole-plant contributions between *C. rhomboidea* and *P. radiata* were not significant (Student's *t*-test, *P* > 0.05 for all comparisons).

The contribution of water from capacitance into transpiration stream should be most impactful during rapid changes in transpiration, such as during the morning transition from dark to light, rather than contribution to total daily usage. Calculating the volume of water discharged from capacitance for each individual revealed that in the first hour of the lights being turned on during a typical day, the theoretical maximum capacitance contribution to the total transpired water of *C. rhomboidea* was 80.3% ± 16.9% SE. This capacitance water was largely from the root capacitor 52.1% ± 11.2% SE, with 28.2% ± 5.8% SE from the above-ground plant (Fig. 4). Under the same circumstances *P. radiata* could draw a theoretical maximum whole plant capacitance contribution of 37.4% ± 7.5% SE for the first hour of transpiration. This 37.4% of capacitance consisted of 23.8% ± 5.2% SE from the roots, and 13.6% ± 2.3% SE from the canopy (Fig. 4).

**Figure 5.**
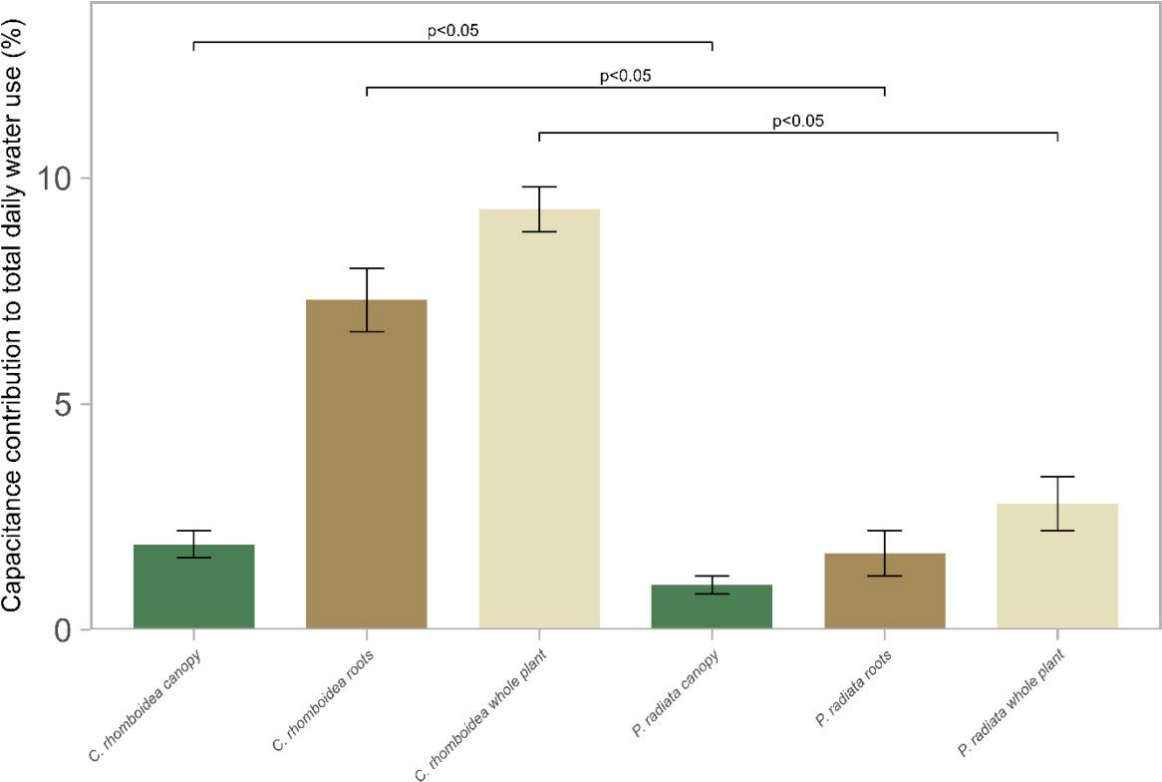
Contribution of root, canopy, and whole-plant water storage to total daily water demand in *C. rhomboidea* and *P. radiata*. Percentage (±SE) of total transpired water sourced from internal storage in the canopy (green), root (brown), and the combination of both (cream) for *C. rhomboidea* and *P. radiata* during an average full day of transpiration in a growth chamber (07:00–17:00 h). Values represent means calculated from *E*_c_ and C measurements (*n* = 3 for both species). Differences in canopy, root, and whole-plant contributions between *C. rhomboidea* and *P. radiata* were significant (Student's *t*-test, *P* < 0.05 for all comparisons).

The mean capacitor contribution to total daily usage in *C. rhomboidea* was 9.3% ± 0.5% SE (canopy 1.9% ± 0.3% SE, roots 7.4% ± 0.7% SE), while in *P. radiata* only 2.8% ± 0.7% SE (canopy 1% ± 0.2% SE, roots 1.7% ± 0.5% SE) of daily transpiration was accounted for by capacitance (Fig. 5).

## Discussion

Here, we have shown root capacitance serves as an important buffer against rapid changes in transpiration in 2 conifer species and provides a substantial source of water at the daily scale in one of these species. The available water stored as capacitance in the roots of the 2 contrasting conifer species was similar to above-ground capacitance in *P. radiata*, and much larger that the above-ground capacitance in *C. rhomboidea*. Furthermore, under the conditions of our study, the water storage reservoirs required the entire night to refill, indicating that the functionality of capacitance was consistent with diurnal charging and discharge.

### Capacitance during rapid changes in transpiration

In our study, root capacitance played a key role as a source of water for transpiration during the hours following the transition from darkness to light in the morning. Theoretically, this would allow plants to sustain higher transpiration rates in the morning than one would expect if water was sourced solely from the soil (Phillips et al. 2003). The smaller resistance to water flowing out of capacitors, compared with high resistance to water flowing into the roots from the soil (Steudle and Peterson 1998) should enable less water potential drop under high transpiration, and therefore allow transiently higher stomatal aperture and photosynthetic rate. Without this parallel supply of up to 50% of transpiration from capacitance during transient increases in demand, the leaf water potential would fall to a point where stomatal closure would be triggered, thereby reducing the maximum attainable assimilation rate (Wong et al. 1979).

The extrapolation of root capacitance role to other species under field conditions is speculative and challenging, primarily due to the scarcity of measurements concerning root capacitance, as well as minimal above-ground capacitance measurements. However, the limited research in this area should not imply that root capacitance is insignificant under natural conditions. For instance, certain species like the subtropical palm tree *S. palmetto* have been observed to rely heavily on internally stored water, with up to 60% of transpired water being sourced from above-ground sapwood (Holbrook and Sinclair 1992). It is possible that adding the unmeasured below-ground component of capacitance may account for most transpired water during transient rises in *E*_c_ coming from stored water.

When root water storage is both substantial and regularly depleted to contribute to the demands of daytime transpiration, it must require a substantial portion of the 24-hour cycle to replenish. This replenishment is presumed to occur primarily at night. Rehydrating the internal water storage over the course of the night can be seen as an efficient use of the water transport tissues. These tissues, which are resource-intensive to construct (Baas et al. 2004), would otherwise remain under-utilized at night. Moreover, by recharging these water reserves at night, plants can begin the day with readily available water for transpiration, bypassing the major resistance in the water transport pathway: the soil-to-root barrier (Goldstein et al. 1998; Peramaki et al. 2001). This is particularly advantageous for roots with high resistance and high capacitances, which may take a whole night to refill and several daylight hours to deplete. It could also be speculated that this strategy could be especially beneficial for gymnosperms. Given that gymnosperms’ water transport is less efficient (greater total resistance to water flow) than that of angiosperms due to differences in vascular tissues (Bond 1989; Brodribb and Feild 2010), these species might benefit disproportionately from high capacitance that could enable higher transpiration rates, and therefore greater productivity. We hypothesize that the water storage in conifers, both below-ground and above-ground, represents an adaptive strategy to mitigate their reliance on a less efficient soil-leaf water transport system compared to angiosperms. Nevertheless, further research is necessary to substantiate this claim, as there is limited existing research on this potential association.

The identification of substantial root capacitance in 2 conifer species raises the question of where water is stored in these tissues. Tissues with living cells with elastic walls, which can undergo substantial volume changes as turgor pressure changes, such as intracellular storage elements (Holbrook, 1995; Tyree and Yang 1990) and parenchyma cells (Holbrook and Sinclair 1992), are highlighted as promising candidates. Despite the limited literature on this subject, there is evidence of diurnal root shrinkage patterns (Huck et al. 1970; Cole and Alston 1974; Faiz and Weatherley 1982; Taylor and Willatt 1983; Nobel and Cui 1992), although these studies did not pinpoint the specific root tissues responsible for the bulk of the shrinkage. Previous attempts to distinguish between the shrinkage behaviors of different root tissues have been confined to analyzing root segments at specific distances from the root tip (Taylor and Willatt 1983) or focusing on seminal roots (Cole and Alston 1974). We argue that the observed root volume changes, through swelling and shrinking, likely contribute to capacitance across diurnal shifts in water potential, mirroring the well documented behavior observed in leaves (Schymanski et al. 2013). In particular the younger, elastic tissues in fine roots (Loades et al. 2015), seem to be a likely location for the storage of large volumes of water.

Understanding how water contribution from root capacitance to the transpiration stream varies across different plant species throughout their life cycles is crucial. The total contribution of water from root capacitance, and the percentage relative to the above-ground plant are presumably heavily dictated by root/shoot ratios, and the total biomass of the below ground plant. However, estimating these values is arduous and destructive, as well as being somewhat plastic (Qi et al. 2019). A methodological review by Mokany et al. (2006) showed shrubland and grassland species typically exhibit a decreasing root-to-shoot ratio as they mature. This potentially indicate a diminished ability to rely upon root water storage. But by contrast, the same review showed that forest and woodland species generally maintain relatively consistent root-to-shoot ratios throughout their life cycles. Moreover, there is evidence suggesting an increase in above-ground biomass is associated with an enhanced above-ground capacitance contribution in a wide range of species (Scholz et al. 2011). Extrapolating net root capacitance contributions as a percentage of water use from saplings to mature trees could also be challenging due to tree age, which can affect root anatomy, physiology, and the hierarchy of root orders. In mature trees, higher root orders have been linked to greater nutrient storage (Peri et al. 2008); however, the relationship between root order and water storage has received little attention.

To measure root and stem capacitance, we adapted a rehydration technique previously used to measure the water storage capacity of leaves (Blackman et al. 2011). This method directly gauges the water storage capacity of the intact root system, providing high accuracy at the cost of being destructive to measure. This contrasts with other studies that have measured the root system by assessing the water storage capacity of selected woody and/or fine root sections, then estimating capacitance from the mass of the entire root system, assuming the capacitance is uniform (Running 1979; Nobel and Jordan 1983; Zhou et al. 2019). Our direct measurement of the entire root system presents a challenge when attempting to compare with other studies, primarily because our measurements are normalized by leaf area. Other root capacitance studies have employed different normalizing factors, such as root weight (Domec et al. 2006) or fresh root tissue area (Running 1979; Nobel and Jordan 1983). We chose to normalize root capacitance by leaf area as our primary interest lies in understanding the contribution of root capacitance to the transpiration stream, where vapor fluxes are typically expressed per leaf area.

Our conclusions are based on experiments with potted plants, understanding that there is some uncertainty about potential plastic responses in root development due to the constraints of being grown in pots. A meta-analysis on the influence of pot size (Poorter et al. 2012) found that changes to root-to-shoot ratios due to pot size are variable, though there is believed to be a small average effect. Due to this it is possible our results could be over or underestimating the ratio of water storage between the above- or below-ground plant relative to wild grown individuals under similar environmental conditions. Further study in field plants is needed to assess the influence of pot volume.

Similar to models that treat the capacitance of stems and leaves as being in parallel when assessing their combined contributions (Tyree 1988), we have modeled the root and canopy capacitance as if they are in parallel, summing their contributions to calculate their role in meeting diurnal transpiration demands. This assumes that water in root capacitance is contained in the cortex as a symplastic volume, parallel with the main apoplastic flow path (Steudle and Peterson 1998).

## Conclusions

Our study demonstrates that the roots of 2 conifer species, particularly *C. rhomboidea*, exhibits a substantial amount of capacitance. This capacitance contributes to meeting the water demands of diurnal transpiration, particularly during transient conditions, highlighting a potentially important source of stored water in plants. Historically, root capacitance has been largely overlooked, yet it may be essential for understanding plant water dynamics, akin to the recognized importance of above-ground capacitance. Further research is needed to determine the prevalence of root capacitance across different species, to assess how environmental fluctuations impact its contribution to daily water usage, and to understand its role throughout a plant's lifecycle.

## Materials and methods

### Plant materials and growth conditions

All measurements were taken using Oyster Bay pine (*C. rhomboidea)* and Monterey pine (*P. radiata)* saplings, grown under unfiltered natural light in glasshouses at the University of Tasmania. All plants were grown in 5-L pots using a native potting mix. All individuals were approximately 2 years old. *C. rhomboidea* plants were on average 80 cm tall, and *P. radiata* plants were on average 70 cm tall.

### Continuous measurements of E*_c_* and Ψ*_stem_*

To assess the possible impact of capacitance on diurnal whole plant gas exchange, we initially characterized the dynamics of transpiration and water potential change in plants under controlled conditions. Continuous measurements of diurnal canopy transpiration (*E*_c_) and Ψ_Stem_ were collected from the 2 species (*n* = 3 for both species) under controlled conditions in a growth chamber (Supplementary Figs. S1 and S2). Individuals were placed in a growth chamber and allowed 3-4 days to acclimatize before the experiment. For all experiments with day/night cycles, environmental conditions were fixed throughout the day with an average temperature of 23.5 °C (±0.88), relative humidity of 40% (±7), and a day length of 9 h, light intensity was fixed at 470 µmol quanta m^−2^ s^−1^ PPFD at the canopy level. Night temperatures averaged 19.5 °C (±0.8), with relative humidity 46% (±9). Control over temperature and humidity was provided using a standard air conditioning unit (5KW Daikin Inverter Split System L-Series FTXS50LVMA, Honshu, Japan). Humidity and temperature were measured using HOBO MX2301A Temperature/RH Data Logger device (Onset, Massachusetts, USA) and light intensity being measured by HOBO Pendant Temp/Light MX2202 Logger (Onset, Massachusetts, USA).

Canopy transpiration rate (*E*_c_) was continuously monitored by placing potted plants of both species on computer-interfaced balances (logging every 5 min) to an accuracy of ± 0.01 g (Mettler Toledo Precision Balance XS6002S or Mettler Toledo Precision Balance ME3002E). Pots were covered with aluminum foil to prevent soil evaporation.

In each plant, *E*_c_ was averaged over 30 min and normalized to projected whole plant leaf area (m^2^) measured at the end of the experiment using a flatbed scanner in combination with ImageJ. For each individual plant, an optical dendrometer (Cavicam Co, Hobart, Australia) (for details see Bourbia et al. 2021, 2022; Bourbia and Brodribb 2023) was attached to a terminal branchlet for both *C. rhomboidea* and *P. radiata* to monitor width variation every 5 min and used to infer dynamics of *Ψ*_stem._ Branchlet width dynamics were calibrated in each individual plant against *Ψ*_stem_ values measured with a Scholander chamber (PMS, Albany, OR, USA). Calibration measurements were performed on nontranspiring neighboring branchlets that were covered with aluminum foil and wet paper towel for at least 1 h before measurements. These measurements were performed over different periods of the day, and over several days to cover most of the range of width observed during measurements. While *Ψ*_stem_ and width were always linear with very high r^2^ (>0.99), the extreme ends of stem water potential values are extrapolated from this linear fit (Supplementary Fig. S3). Other studies, such as (Bourbia and Brodribb 2024), have observed that this linear relationship in *C. rhomboidea* extends beyond the calibrated ranges used in the present study.

### Ψ_stem_ recovery with and without a root system

To assess whether roots provide capacitance water to the canopy, 3 well-hydrated plants of both species were kept under high humidity conditions overnight to ensure each plant was as hydrated as possible. The following day, while keeping the canopy of each potted plant covered to prevent transpiration, soil was gently removed from the root system, leaving each plant with exposed roots. Afterward, the root system of each plant was gently patted with a paper towel to remove excess water. For each individual plant, an optical dendrometer was fitted to either a leaf (*P. radiata*) or branchlet (*C. rhomboidea*) to measure *Ψ*_stem_, using the same method detailed above. Then, each plant was left overnight with the leaves covered with damp paper towel to limit nocturnal transpiration. The roots were directly covered with dry cloth, and then surrounded with an outer layer of damp paper towels to prevent transpiration from the roots, allowing the plant to reach a stable *Ψ*_stem_. Under such conditions, changes in *Ψ*_stem_ can be attributed to the redistribution of water from upstream of the leaves toward the monitored leaf tissue. The following day, plants were then either left with their root systems attached (3 plants) or had the roots cut off (3 plants) from the stem near the root collar, and the site of the cut covered with petroleum jelly to prevent water movement out of the cut. The canopy of plants was then exposed to daylight to transpire until *Ψ*_stem_ reached a value comparable to that of a standard end-of-day *Ψ*_stem_, which was monitored using an optical dendrometer. Once this state has been reached, plants were then covered again as described before to assess if the *Ψ*_stem_ recover using water sourced from internal water reservoirs. Recovery was calculated as the maximum *Ψ*_stem_ restored using only internal water storage after a period of 12 h in darkness, relative to the difference between the maximum *Ψ*_stem_ (measured before soil-less dehydration) and the minimum *Ψ*_stem_.

### Capacitance

Using the same 3 plants per species that had been monitored in the growth chambers (see above) separate capacitance measurements were made for the above-ground and below-ground tissue. Each plant was removed from their pots, and then washed gently to remove all soil from the roots. Plants were then fitted with an optical dendrometer on a branchlet to monitor Ψ_Stem_, then dried slowly (attempting to approximate normal daytime pressure dynamics) using alternating light and dark periods of around 8 h, until reaching a Ψ_Stem_ slightly below the lowest diurnal value recorded under diurnal chamber conditions (approximately −1 mPa). Whole plants were then covered by a wet plastic bag to stop residual transpiration and enabling Ψ_Stem_ equilibrium within the plant, which was indicated when the optically derived Ψ_Stem_ had plateaued for at least 2 h. Plants were then cut ∼2 cm above the root collar, separating the above-ground and below-ground tissues.

Each of the 2 plant sections were recut under water and then connected to tubing that allowed the 2 parts of plant tissue to pull water from a beaker sitting on a computer-interfaced balance (Mettler Toledo MS204S/01, ± 0.0001 g), which was weighed continuously at 5 s intervals to allow a measurement of water flow into each tissue segment. After 8 h, the length of a typical night, a final Ψ_Stem_ measurement was taken of the roots, and a separate net capacitance of the above-ground and below-ground plant was estimated as follows:


(1) $$C=\frac{m_{f}-m_{i}}{\Delta \Psi }$$

where *C* is capacitance expressed as mmol m^−2^ MPa^−1^, $m_{f}$, and $m_{i}$ are final mass of water after rehydration and initial mass before rehydrating in mmol and normalized to leaf area, ΔΨ is the change in water potential during rehydration.

During rehydration, leaves were covered with damp paper towels to prevent transpiration. Transpiration from roots was similarly eliminated during rehydration by ensuring that the roots were covered with dry cloth and surrounded with wet paper towels but were deliberately not allowed to directly touch the wet paper towels to prevent possible uptake of water. The ΔΨ was taken from the difference between the initial Ψ_Stem_ measured from equilibrated plants before rehydration minus the average Ψ_Root_ of ∼15 roots measured after rehydration using a Scholander chamber (PMS, Albany, OR, USA). The sampled roots deliberately represented the entire range of root sizes, and distance to the stem. To compare between the 2 species, capacitance values were normalized to canopy surface area (Supplementary Table S1).

### Estimating capacitance contribution to daily transpiration

The flow rate of water from a hydraulic capacitor can be described using the analogous system of RC circuit (Brodribb and Holbrook 2003; Blackman et al. 2011). By knowing the net root system capacitance, and canopy system capacitance, we can calculate the conductances of the respective tissue segments (2).


(2) $$K_{rc}=\left(C\times \ln\;\left(\frac{\Psi_{i}}{\Psi_{f}}\right)\right)/t$$

where *K*_rc_ is either the canopy or root hydraulic conductance as mmol m^−2^ MPa^−1^ s^−1^ (Supplementary Table S2), *C* is either the canopy or root capacitance as mmol m^−2^ MPa^−1^, $\Psi_{i}$ is the initial Ψ  _root/stem_ before rehydrating, $\Psi_{f}$ is the final Ψ  _root/stem_ after rehydrating for *t* seconds.

By having separate approximations for *C* and *K*_rc_, both measured on plants after removal from the soil (as described above) of the 2 species, we applied these *C* and *K*_rc_ measurements to the same individual plants under typical conditions in a growth chamber. To estimate total contribution to daily transpiration using (4), we calculate the initial flow rate from the capacitor solved by (3).


(3) $$F_{0}=\Delta \Psi \times K_{\mathrm{rc}}$$

where $F_{0}$ is flow as mmol s^−1^, ΔΨ is the difference in Ψ_Stem_ as MPa at maximum rehydration occurring at the beginning of the day and the lowest point during the end of the transpiration period, and *K*_rc_ is the conductance from the root to capacitor as mmol m^−2^ s^−1^ MPa^−1^.

By now knowing a calculated value for both the initial flow and the *K* of both the above-ground and below-ground plant segments, we can use (4) to estimate the volume of water moving from either capacitor to the xylem through the t second from the beginning of the day with a known ΔΨ  _stem_, and hence capacitance contribution to daily transpiration.


(4) $$\mathrm{Mass}\;\mathrm{of}\;\mathrm{water}\;\mathrm{moving}\;\mathrm{from}\;\mathrm{capacitor}\;\mathrm{to}\;\mathrm{xylem}\;=\overset{f}{\underset{i}{\int }}\;F_{0}\times e^{\frac{-kt}{c}}$$

where *t* is the period of interest in seconds, *k* represents the hydraulic conductance of either tissue segment as mmol m^−2^ MPa^−1^ s^−1^, and *c* represents the capacitance of either tissue segment as mmol m^−2^ MPa. For the total daily contribution, a *t* of 9 h of daytime transpiration was used, while the contribution to the first hour of daytime transpiration was also calculated. The mass of water moving from capacitor to xylem was expressed as a fraction of the cumulative *E*_c_ over the period of interest. Contribution of water from capacitors during both the 1 and 9-hour time periods assume the capacitors are discharging water only and not recharging due to the continuous water potential drops over these periods under these study conditions.

### Data analysis

We used linear regressions to quantify the correlation between branchlet width variation and Ψ_Stem_. Differences in the mean values of capacitance between the 2 species and between the above- and below-ground parts of the plant were tested with Student's *t*-tests after testing for normality and homogeneity of variances. Results are presented as mean values of ± SD, unless otherwise stated as ± SE for estimated values. Differences were significant when *P* < 0.05. Figures were created using R version 3.5.3. Calculations of leaf area were done using ImageJ version 1.53t.

## Supplementary Material

kiaf116_Supplementary_Data

## Data Availability

Background data such as time-series detail are available upon request to the corresponding author.

## Supplementary data

The following materials are available in the online version of this article.

**Supplementary Figure S1.** Vapor pressure deficit (VPD) in kPa inside the growth chamber during data collection for Fig. 1.

**Supplementary Figure S2.** Temperature inside the growth chamber during data collection for Fig. 1.

**Supplementary Figure S3.** Calibration for the optical dendrometer.

**Supplementary Table S1.** Table listing species names and plant numbers, with the respective leaf areas of the plants.

**Supplementary Table S2.** Table listing species names and plant numbers, along with conductance values.

## Dive Curated Terms

The following phenotypic, genotypic, and functional terms are of significance to the work described in this paper:

stem AmiGo: PO:0009047

water CHEBI: CHEBI:15377

whole plant AmiGo: PO:0000003
