# Supplementary material for: Substantial capacitance found in the roots of 2 contrasting conifer species
Source: Plant Physiol. 2025 May 7;198(1):kiaf116. doi: 10.1093/plphys/kiaf116 (PMC12056505; doi:10.1093/plphys/kiaf116)
Supplement: kiaf116_Supplementary_Data [file kiaf116_supplementary_data.pdf]

Supplementary Data

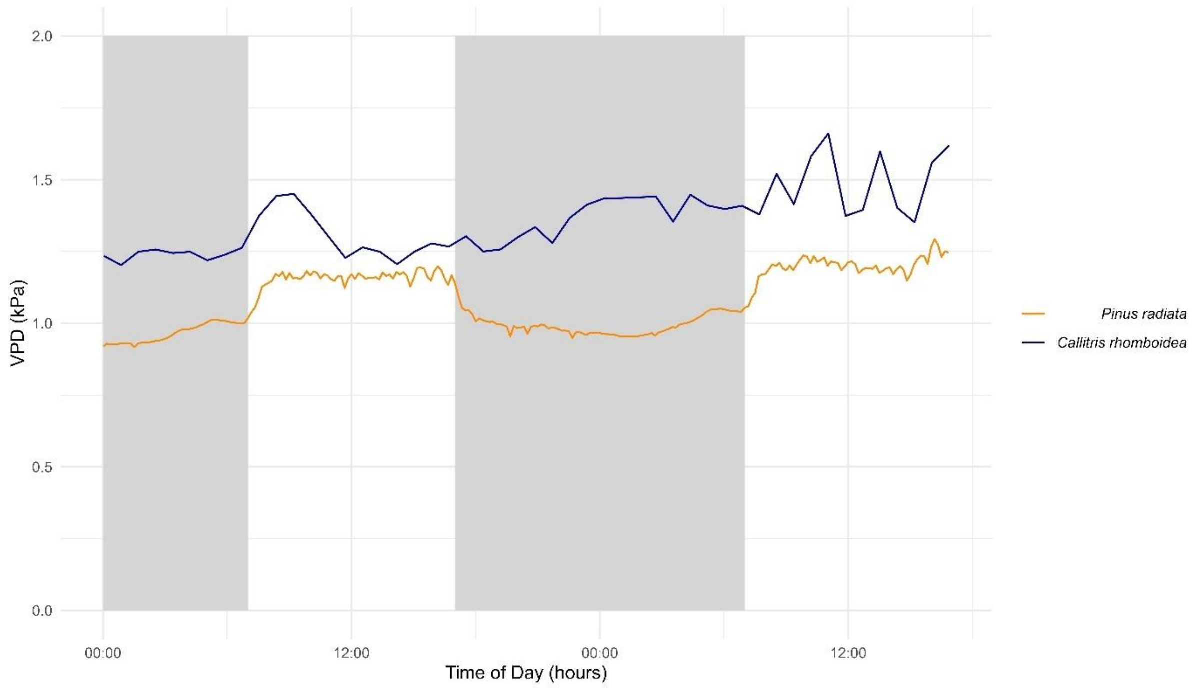

Supplementary Figure S1 Vapor pressure deficit (VPD) in kPa inside the growth chamber during data collection for Fig. 1. Gray periods indicate when lights were turned off. The orange line represents the VPD conditions for all three *Pinus radiata*, while the blue line represents all three *Callitris rhomboidea*.

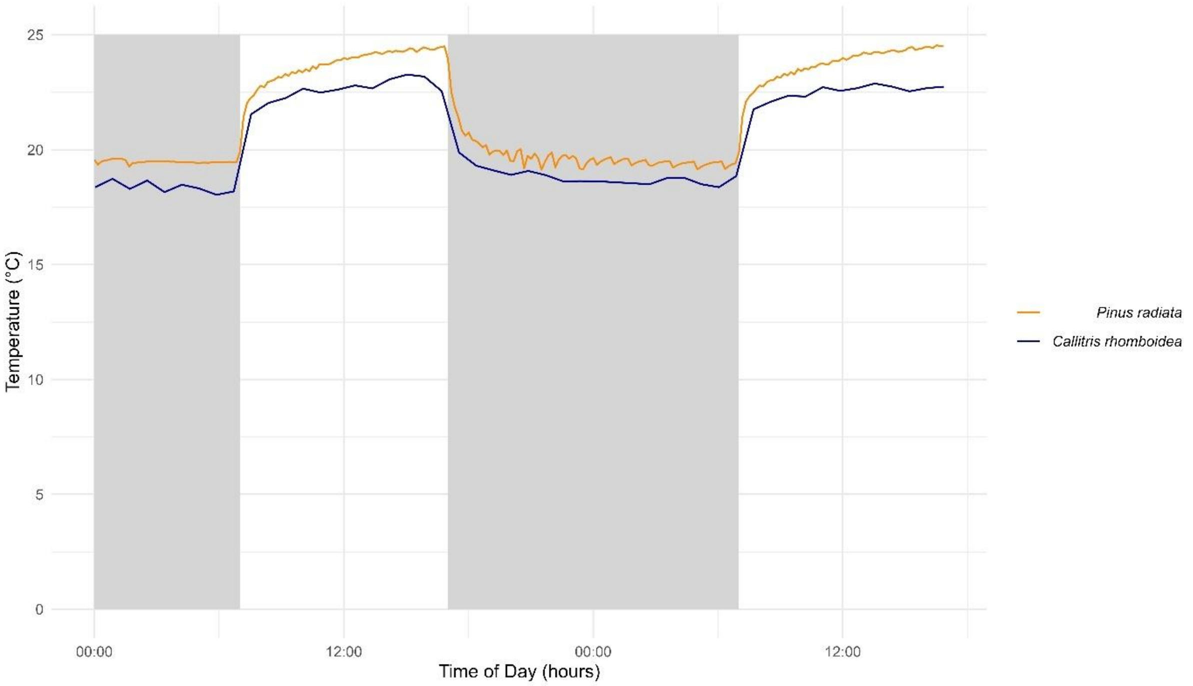

Supplementary Figure S2 Temperature inside the growth chamber during data collection for Fig. 1. Gray periods indicate when lights were turned off. The orange line represents the temperature for all three *Pinus radiata*, while the blue line represents all three *Callitris rhomboidea*.

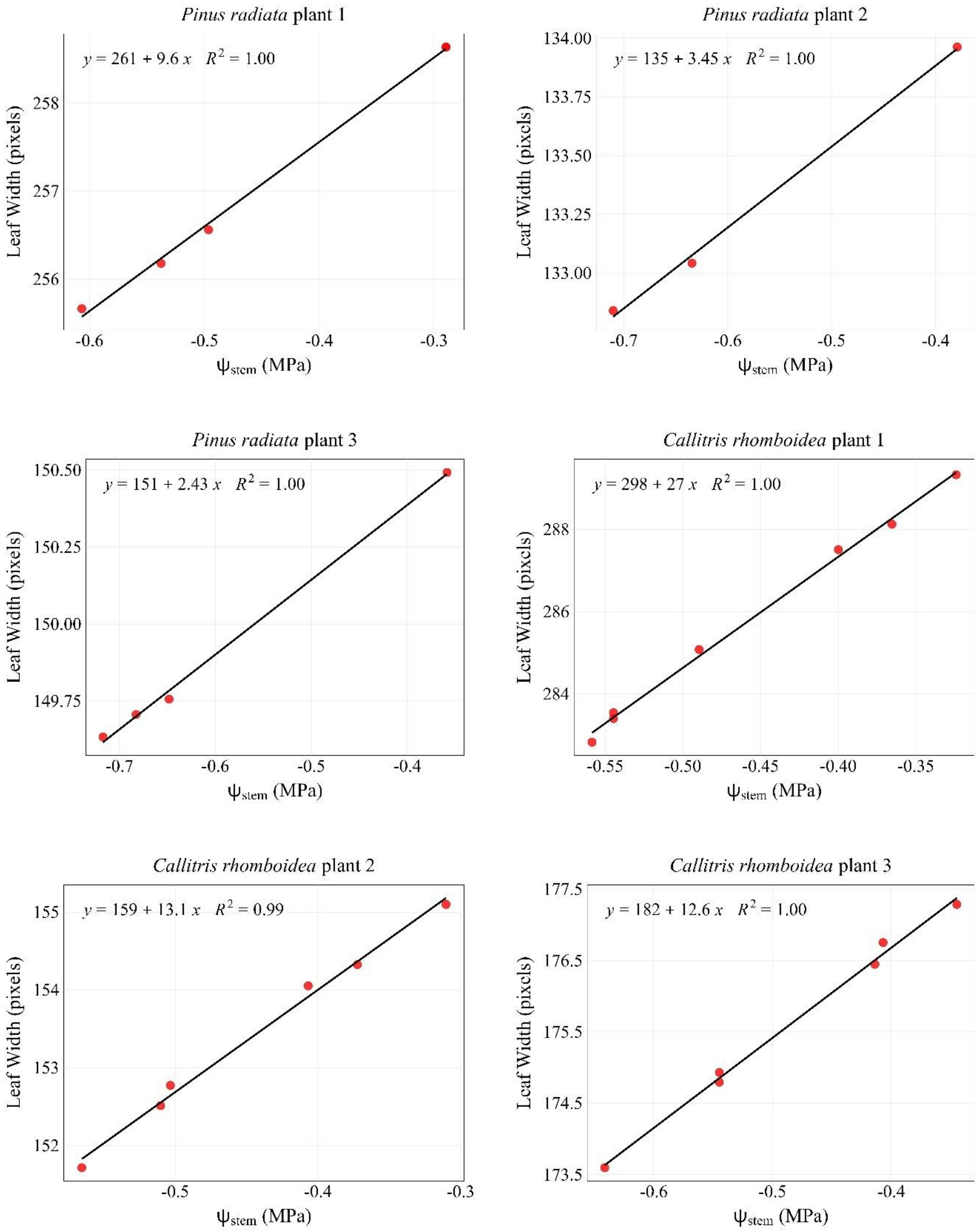

Supplementary Figure S3 Calibration for the optical dendrometer. Each red dot represents the intersection of Ψ_{stem} measurements using a Scholander chamber (as MPa), and a leaf width recorded by an optical dendrometer of a neighboring leaf/branchlet.

Supplementary Table S1 Table listing species names and plant numbers, with the respective leaf areas of the plants. Leaf area used to normalize typical diurnal water relations and above- and belowground capacitance.

Species	Plant Number	Leaf Area (m^2)
Callitris rhomboidea	1	0.058322
Callitris rhomboidea	2	0.048631
Callitris rhomboidea	3	0.062864
Pinus radiata	1	0.087286
Pinus radiata	2	0.105313
Pinus radiata	3	0.102244

Supplementary Table S2 Table listing species names and plant numbers, along with conductance values. Two conductance values are presented: one from the cut root collar to the canopy (as mmol m^{-2} MPa^{-1} s^{-1}), and one from the cut root collar to the root system (as mmol m^{-2} MPa^{-1} s^{-1}).

Species	Plant Number	Canopy conductance (mmol m^{-2} MPa^{-1} s^{-1})	Root conductance (mmol m^{-2} MPa^{-1} s^{-1})
Pinus radiata	1	1.93	2.56
Pinus radiata	2	1.52	1.15
Pinus radiata	3	2.24	3.27
Callitris rhomboidea	1	3.86	2.34
Callitris rhomboidea	2	3.33	2.40
Callitris rhomboidea	3	4.13	3.26
